# Evaluation of clinical outcomes of raised intraocular pressure following intravitreal triamcinolone acetonide injection

**DOI:** 10.22336/rjo.2024.08

**Published:** 2024

**Authors:** Pragya Singh, R Krishnaprasad, Guruprasad Ayachit, Shrinivas Joshi

**Affiliations:** * MM Joshi Eye Institute, Karnataka, India; ** Department of Glaucoma, MM Joshi Eye Institute, Karnataka, India; *** Department of Vitreoretina services, MM Joshi Eye Institute, Karnataka, India

**Keywords:** intraocular pressure, intravitreal triamcinolone acetonide injection, steroid-induced glaucoma, anti-glaucoma medications

## Abstract

**Aim:** To assess the incidence, risk factors, and treatment outcomes in intravitreal triamcinolone acetonide injection (IVTA) induced intraocular pressure rise and to compare IOP rise in 1-mg and 2-mg IVTA.

**Materials and methods:** Prospective observational study conducted in all eyes receiving IVTA. Any pre-existing glaucoma and patients who received IVTA or dexamethasone implant in the last 6 months were excluded.

**Results:** 9 between 61-70 years of age developed an IOP spike. The mean and standard deviation of age in years was 61.95 ± 8.70. Maximum eyes had ME due to Diabetic Retinopathy (53.3%). All cases of uveitic ME were reported to have an IOP spike. 2 out of 3 high myopic eyes and 1 eye with thyroid abnormality had an IOP spike. High IOP was found in 13 eyes, with more than 25 mm Hg rise in 4 eyes and more than 5 mm Hg rise from baseline IOP in 9 eyes. The mean and standard deviation of time taken for IOP raise (in days) was 46.39 ± 37.68. A total of 38 eyes received 1 mg of IVTA and the rest 22 received 2 mg of IVTA. 23.7% of 1 mg eyes experienced an IOP rise while it was 18.2% in eyes with 2 mg IVTA. The injection was repeated in 12 eyes and 41.7% developed an IOP spike among them. The independent “t” test results showed that there was a significant difference in the mean of IOP (Pre-injection) concerning the IOP rise (P=0.007*). 1 eye had IVTA crystals in the anterior chamber with raised IOP of 30 mm Hg. 1 out of 13 eyes with raised IOP needed 2 AGMs, the other 12 eyes responded well to 1 AGM.

**Discussion:** IVTA is widely used in refractory cases of ME and steroid-induced glaucoma is the most common side effect of IVTA. To the best of our knowledge, there is a lack of literature on prospective studies on IVTA-associated risk factors, patterns of IOP elevation, and treatment outcomes. The pre-injection mean ± SD baseline IOP for uneventful eyes was 12.87±2.65 and the pre-injection mean IOP for eyes with IOP event was 15.23±2.89 (P=0.007*).

**Conclusion:** We proposed that TA is an independent risk factor for post-intravitreal injection IOP spike. IVTA causes a maximum IOP spike at 1 to 2 months and has a protracted course that responds to anti-glaucoma medications. High baseline IOP, a repeated dose of IVTA, the presence of TA crystals in the anterior chamber, and high myopia were associated with significant IOP elevation.

**Abbreviations:** ACD = Anterior chamber depth, AS = Anterior segment, AGM = Anti-glaucoma medications, ARMD = Age-related macular degeneration, BCVA = Best-corrected visual acuity, BRVO = Branch retinal vein occlusion, CCT = Central corneal thickness, CRVO = Central retinal vein occlusion, CME = Cystoid macular edema, CNVM = Choroidal neovascularization membrane, CSME = Clinically significant macular edema, DR = Diabetic retinopathy, ERM = Epiretinal membrane, IOP = Intraocular pressure, IGS = Irvine-Grass syndrome, GAGs = Glycosaminoglycans, IVTA = Intravitreal triamcinolone acetonide injection, ME = Macular edema, NVG = Neovascular glaucoma, OHT = Ocular hypertension, PDS = Pigment dispersion syndrome, PACG = Primary closed angle glaucoma, POAG = Primary open-angle glaucoma, PXF = Pseudoexfoliation, VA = Visual acuity, VEGF = Vascular endothelial growth factors, VH = Vonherick’s grading, SD = Standard deviation, TA = Triamcinolone acetonide, TIGR = Trabecular meshwork inducible glucocorticoid response

## Introduction

Corticosteroid-induced glaucoma was first described in the 1950s [**1**-**3**]. Intravitreal dexamethasone was the first intravitreal steroid injection performed for the treatment of endophthalmitis by Graham and associates in 1974 [**4**]. Triamcinolone acetonide (TA) is a synthetic steroid of the glucocorticoid family, available as an ester, and due to its hydrophobic nature, it maintains vitreous levels for up to 3 months [**5**-**7**] but can have maximum effect until 140 days [**5**]. It is the most commonly used intraocular steroid as its fibroblast growth inhibition [**6**].

Even after proven efficacy of various anti-vascular endothelial growth factors, intravitreal triamcinolone acetonide (IVTA) is being widely used in refractory cases of macular edema (ME) secondary to diabetic retinopathy (DR), central retinal vein occlusion (CRVO), branch retinal vein occlusion (BRVO), uveitis, Irvine-Grass syndrome (IGS), exudative age-related macular degeneration (ARMD) [**8**], sympathetic ophthalmia, cystoid macular edema (CME), Vogt-Koyanagi-Harada syndrome [**9**]. IOP rise following IVTA occurs in 40% [**10**] to 50% [**11**] of cases depending on dose and duration of follow-up. Steroid-induced glaucoma is the most common side effect of IVTA [**8**].

Steroid causes chronic open-angle glaucoma by various mechanisms: (1) accumulation of basement membrane-like material, staining for type IV collagen causes physical and mechanical changes in the microstructure of trabecular meshwork [**1**,**2**,**11**]; (2) increased deposition of glycosaminoglycans (GAG), polymerized GAGs become hydrated, producing biologic edema and outflow resistance [**1**,**11**]; (3) decreased breakdown of substances in the trabecular meshwork [**1**,**2**,**11**]; (4) production of the trabecular meshwork inducible glucocorticoid response (TIGR) protein inhibiting aqueous outflow [**1**,**2**,**12**]; (5) crystalline steroid particles can block trabecular outflow [**1**,**13**]; (6) FKBP06-binding immunophilin FKBP51 mediates nuclear transport of the human glucocorticoid receptor beta alter the trabecular meshwork cell morphology [**1**,**2**].

Risk factors for IVTA-induced glaucoma are pre-existing glaucoma (primary open angle glaucoma (POAG) or ocular hypertension (OHT)), family history of glaucoma, age (bimodal distribution peaking at age 6 and old age at the highest risk), connective tissue disease, diabetes, high myopia [**9**] eyes with pigment dispersion syndrome (PDS) or traumatic angle recession [**1**]. 1 mg dose of IVTA has the least incidence of IOP rise and lenticular opacity [**14**]. So, the identification of risk factors contributing to IOP rise has become important to prevent glaucomatous damage. Treatment with anti-glaucoma medications (AGM) usually controls IOP in most patients and AGM can be discontinued within 6 months [**15**]. Surgical management is needed in less than 2% [**16**]. This study was conducted to assess the incidence, risk factors, and treatment outcomes of IVTA-induced IOP rise and its relationship with TA dose.

## Materials and methods

A prospective observational study was conducted on 60 eyes of 58 patients receiving IVTA at MM Joshi Eye Institute, Hubli, from December 2018 to May 2019. Written informed consent was obtained.

Parameters noted were: duration and type of diabetes, lens status pseudophakic/phakic/aphakic, history of myopia/hypertension/thyroid diseases/connective tissue disorders/ hypercortisolism, family history of glaucoma, dose of IVTA – 1 mg or 2 mg (decided by randomization), etiology of ME, repeated IVTA injections.

Parameters noted pre-injection and during each follow-up: visual acuity (VA), best-corrected visual acuity (BCVA), slit lamp examination for anterior segment (AS), anterior chamber depth (ACD) - based on Van Herick’s grading, corneal thickness corrected IOP using Goldmann Applanation Tonometry were recorded. Gonioscopy using a 4-mirror gonio lens Sussman model, slit lamp biomicroscopy for vertical cup disc ratio, neuroretinal rim and retinal nerve fiber layer defects in undilated pupil followed by dilated fundus evaluation by the same observer.

*Inclusion criteria*

All eyes receiving IVTA, including eyes that have received anti-VEGFs before 6 months.

*Exclusion criteria*

1. Pre-existing glaucoma - POAG/primary closed angle glaucoma (PACG)/OHT, glaucoma secondary to trauma, neovascular glaucoma (NVG), PDS, pseudoexfoliation (PXF) and other secondary causes.

2. Raised IOP (> 25 mm Hg) not recorded in previous visits.

3. Patient with peripheral iridotomy/already on AGM/eyes post trabeculectomy/valve surgery. 

4. Patients who have received IVTA (either 1 mg/2 mg) or dexamethasone implant in the last 6 months. 

5. Disc anomalies like tilted/colobomatous discs.

6. Vitrectomized and silicon oil-filled eyes.

Preservative-free TA (Aurocort) comes in a 40 mg/ml vial and was injected as 1 mg or 2 mg.

Follow-up was done post-injection on day 1 and then monthly up to 6 months.

We defined post-IVTA glaucoma as raised IOP above 25 mm of Hg [**17**] or 5 mm Hg [**8**] more than baseline after IVTA injection. So, eyes with more than 25 mm Hg IOP or > 5 mm Hg of IOP rise from baseline were started on AGM, and IOP for such eyes was recorded again after 7 days. Target IOP had to be kept below 15 mm Hg. AGMs were started in the order of the following preference: topical beta-blockers were added as 1st line of treatment (only if no systemic contraindication), followed by topical alpha-adrenergic agonist, followed by carbonic anhydrase inhibitors (topical followed by oral). Prostaglandin analogues were reserved for refractory cases as they are known to potentiate macular edema. For raised IOP > 25 mm Hg or > 5 mm Hg from baseline - a single drug (Topical beta-blockers) was used. When target IOP was not achieved within 1 week, drugs like topical beta-blockers and alpha-adrenergic agonists were used.

The minimum sample size calculated was 57 (P=0.18). The data was collected and entered in the Microsoft Excel sheet. The statistical analysis was done using the Statistics software SPSS 16.0. Randomization was done by computer-generated random numbers. Continuous data was summarized as Mean ± SD (standard deviation) while discrete (categorical) data was in number and percentage. Quantitative data was analyzed by mean, SD, Unpaired t-test, and Karl Pearson correlation. Qualitative data was analyzed by percentage, Chi-square test, and Fisher exact test. P>0.05 was not significant, <0.05 was significant, and P<0.01 was highly significant.

## Results

A total of 60 eyes of 60 patients were studied, 35 right and 25 left eyes. 9 out of 28 eyes of patients between 61-70 years of age developed an IOP spike, followed by 4 out of 24 eyes of patients in the age group of 50-60 years and 1 out of 5 eyes of patients > 70 years of age developed IOP spike. None of the 3 eyes with age < 50 years developed an IOP event. The mean standard deviation of age in years was 61.95 ± 8.70. IOP spike was noted in 4 (22.2%) out of 18 females and in 9 males out of 42.

40 patients were diabetic and 20 had no DM. Most of the patients, i.e. 37, were Type 2 diabetics, out of which 6 developed IOP spikes, while 2 out of 3 Type 1 diabetics developed IOP spikes. The mean and standard deviation of the duration of DM was 7.07 ± 8.29 years. 5 patients in the non-diabetic group had raised IOP, while 15 had normal IOP. A total of 25 eyes belonged to hypertensive patients. Out of 25 eyes, 5 eyes reported raised IOP, while 20 maintained normal IOP.

Most of the eyes were phakic, i.e. 30, 20 eyes were pseudophakic and 10 eyes were aphakic. Maximum IOP rise was noted in 8 phakic eyes, followed by pseudophakic (4), while only 1 aphakic patient had raised IOP. 

**Fig. 1:**
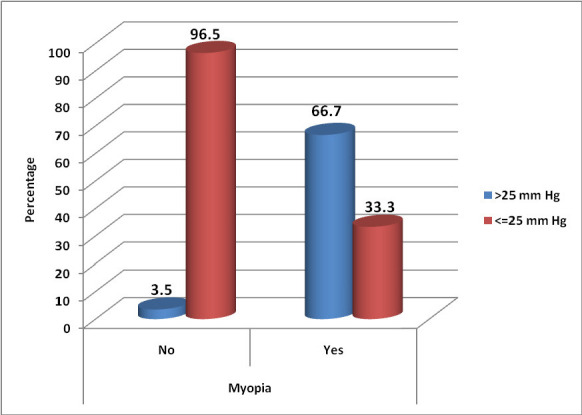
Correlation between Myopia and IOP rise considering > 25 mm Hg

2 out of 3 myopic eyes reported an IOP spike of more than 25 mm Hg, while only 2 non-myopic eyes had an IOP of > 25 mm Hg (P value = < 0.001) (**Fig. 1 A**). 2 myopic and 11 non-myopic eyes had IOP, both > 25 mm Hg and > 5 mm Hg from baseline (P value =0.05).

1 patient had a history of thyroid disease and was reported to have raised IOP of > 25 mm Hg and > 5 mm Hg from baseline. The correlation between thyroid status and IOP spike considering > 25 mm Hg was significant (P value = < 0.001) (**Table 1**).

**Table 1:** Correlation between thyroid status and IOP rise considering > 25 mm Hg

Thyroid disease	IOP rise in follow-ups	
	> 25 mm Hg	< = 25 mm Hg
absent	3 (5.1%)	56
present	1 (100%)	0

**Fig. 2:**
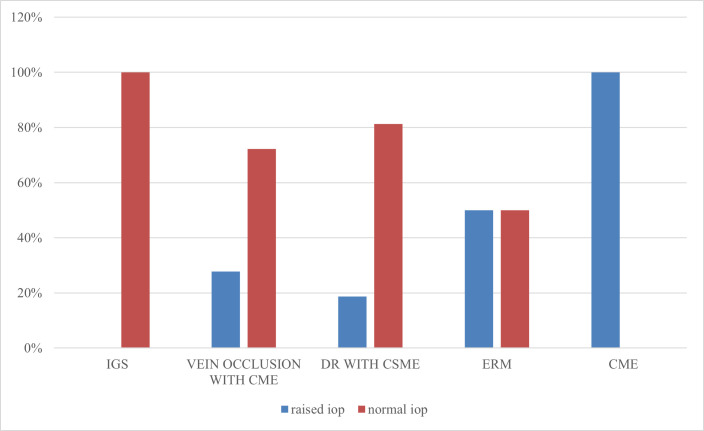
Correlation between retinal pathology and IOP rise

32 (53.3%) eyes were diabetic eyes with CSME, out of which 6 had IOP spike followed by eyes with vein occlusion (**Table 2**). None of the IGS eyes showed raised IOP out of 5, while all uveitic eyes included in the study were reported to have raised IOP (**Fig. 2**). None of the patients had a history of hypercortisolism or connective tissue disorder or family history of glaucoma.

**Table 2:** Retinal pathology

Retinal Pathology	No. of Eyes	Percentage
IGS	5	8.3%
VEIN OCCLUSIONS WITH CME	18	30%
DR WITH CSME	32	53.3%
ERM	2	3.3%
CNVM	1	1.7%
UVEITIS WITH ME	2	3.3%
TOTAL	100	100%

**Fig. 3:**
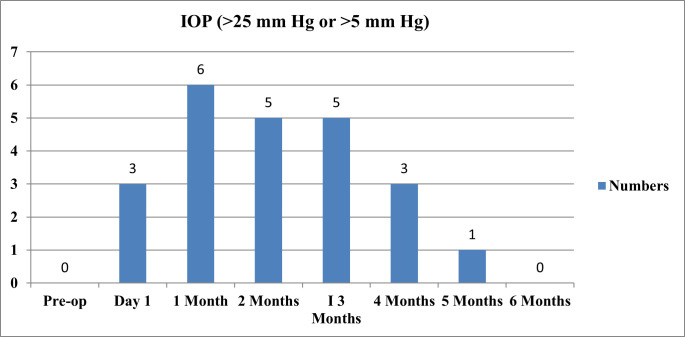
IOP rise concerning time

4 eyes had IOP > 25 mm Hg and 9 had IOP > 5 mm Hg from baseline. 1 eye had an IOP spike of 30 mm Hg. Overall, 13 (21.7%) of the eyes had CCT-corrected raised IOP, while the other 47 (78.3%) had IOP within the normal range. The mean and standard deviation of time taken for IOP rise (in days) was 46.39 ± 37.66. A maximum IOP spike was noted at the end of 1 month (**Fig. 3**).

Both 1 mg and 2 mg groups were matched for age, sex, prevalence and duration of diabetes, hypertension, baseline BCVA, baseline IOP, ACD, cup: disc ratio, and retinal nerve fiber layer. 38 eyes received 1 mg and 22 received 2 mg of IVTA. In the 1 mg group, 9 (23.7%) eyes were reported to have raised IOP, while only 4 (18.2%) eyes were in the 2 mg group. The maximum IOP recorded in the 1 mg group was 24 mm Hg, while 26 mm Hg was recorded in the 2 mg group. 12 eyes received a repeated dose of IVTA. With a single dose, the maximum IOP recorded was 26 mm Hg and repeated IVTA dose had 30 mm Hg as the maximum IOP. Out of 40 eyes that received a single dose, raised IOP was reported in 8 patients, and in the repeated IVTA group 7 patients had normal IOP. The incidence of the IOP spike among repeated doses of IVTA was 41.7%, which was more than the IOP spike among eyes with a single dose of IVTA at 16.7%. The mean IOP in 13 eyes within the raised IOP group was 15.23 ± 2.90, while in the other 47 eyes, the mean IOP was 12.87 ± 2.65. The independent “t” test results showed that there was a significant difference in the mean of baseline IOP of normal eyes with eyes with IOP rise (P=0.007*). The relation between baseline IOP until 6 months in the 1 mg and 2 mg group is shown in **Table 3**.

**Table 3:** Relation between baseline IOP until the 6th month in 1 mg and 2 mg group

IOP	IVTA Dose				
	1mg		2mg		TOTAL
	Mean	SD	Mean	SD	MEAN ± SD
Pre-op	13.50	2.628	13.18	3.261	13.383 ± 2.853
Day 1	14.68	3.418	14.95	3.671	14.783 ± 3.484
1 Month	15.34	4.049	15.27	4.061	15.317 ± 4.019
2 Months	14.71	3.416	14.41	3.621	14.6 ± 3.465
3 Months	14.47	4.059	14.41	3.800	14.45 ± 3.933
4 Months	14.11	3.290	13.77	2.827	13.983 ± 3.104
5 Months	13.486	3.0968	12.964	3.9024	13.292 ± 3.396
6 Months	13.216	2.1620	12.691	3.9384	13.02 ± 2.93

The mean preinjection ACD was 3.73 ± 0.45 and no significant post-injection ACD changes were observed. 13 eyes were categorized to have VH grading III and the rest 47 had VH grading IV. 1 eye out of 13 with raised IOP showed IVTA crystals in the anterior chamber with IOP spike of 30 mm Hg. This eye had a repeated dose of injection and that was statistically significant (P value=0.04).

The mean ± SD for VA pre-injection was 0.72 ± 0.36, which improved to 0.6 ± 0.35 at the end of 6 months. All 60 eyes in our study were open angles and no significant change was noted in subsequent follow-up on gonioscopy. Mean preinjection optic disc cupping was 0.33 ± 0.09 and at the end of 6 months was 0.33 ± 0.10.

12 eyes were administered 1 AGM, while 1 eye used 2 AGMs for 48 ± 26.83 days and 55.71 ± 32.07 days, respectively. Target IOP was achieved at the end of 1 week after starting AGM. None of the eyes needed 3 AGMs or surgical intervention.

## Discussion

IVTA is widely used in refractory cases of ME and steroid-induced glaucoma is the most common side effect of IVTA. Raised IOP needs comprehensive examination, so that intervention at an appropriate time can bring down the rates of IVTA-induced glaucoma. To the best of our knowledge, there is a lack of literature on prospective studies on IVTA-associated risk factors, patterns of IOP elevation, and treatment outcomes. We have tried to exclude all the confounding factors to the extent possible like eyes with pre-existing glaucoma, POAG, PACG, OHT, NVG, PXF, PDS, any steroid use in the last 6 months, trabeculitis, post retina surgery to study the effect of IVTA alone on IOP.

We noted 9 out of 28 eyes of patients between 61-70 years of age to have raised IOP. The higher occurrence of IOP rise among the 61-70 years of age group was probably because ME was more common in this group as we observed mean ± SD for age as 61.95 ± 8.70 years, with 62 years median age, minimum age being 28 and the maximum age being 81 years. Cansu Yuksel-Elgin et al. [**18**] reported older age to have a greater IOP rise and the average age of subjects to be 62.89 ± 7.31 years, which was comparable with our study. 

We observed an IOP spike in 4 out of 18 females, while 9 males out of 42 developed an IOP spike. In previous studies, Cansu Yuksel-Elgin et al. [**18**] reported female gender as a risk factor, but Park et al. [**6**] did not report any correlation between gender and IOP spike.

32 eyes had macular edema due to DR (53.3%), followed by 18 eyes with vein occlusions, 5 eyes with IGS, 2 eyes each for ERM and uveitis with ME, while 1 case had edema due to CNVM. Both eyes with uveitis had an IOP spike, probably due to structural damage in trabecular meshwork induced by previous trabeculitis, but we need a bigger sample size to establish such a correlation. 

Out of all eyes with vein occlusions, 27.78% of eyes showed an IOP spike. Poor ocular perfusion and hypercoagulability are risk factors for POAG and normal tension glaucoma, i.e. high intraocular pressure itself, potentiates vein occlusion [**19**]. We hypothesized that by adding TA we were probably lowering the threshold for IOP rise in eyes with vein occlusion.

Eyes with DR reported an IOP spike in 18.75% of eyes. 1 out of 2 ERM eyes and none of the IGS eyes had an IOP spike. With the chi-square test, none of these etiological factors showed any significant association with IOP rise (p=0.14). Similar to our study, Karakahya et al. proposed that etiology does not cause a significant difference in IOP spike. However, greater IOP rise in the uveitic group [**2**,**20**] and eyes with vein occlusion [**2**] have been reported in previous studies.

The majority, i.e. 61.7%, had Type 2 DM. 2 out of 3 eyes with type 1 DM showed an IOP spike, whereas in type 2 DM 6 out of 37 eyes had a spike. 5 out of 20 non-diabetic eyes were noted to have high IOP. Regarding age, the mean and standard deviation of the duration of DM in our study was 7.07 ± 8.29. A higher incidence of type 1 DM might relate to a longer duration of DM. Reza Razeghinejad M et al. [**2**] proposed a higher incidence of Type 1 DM. A higher incidence in diabetics could be due to a greater number of eyes with DM included in our study. Ansari EA et al. [**21**] proposed a higher IOP spike in diabetics, but Park et al. [**6**] and Wilkin Parke D et al. [**22**] did not report such correlations. We observed that the total eyes of patients with hypertension were 41.7%, out of which 20% developed raised IOP. To evaluate such a higher incidence in hypertensive eyes, we needed to eliminate confounding factors like oral B-blocker use, which might mask the effects of IOP rise. Park et al. also denied a correlation between hypertension and raised IOP [**6**].

We observed 2 high myopic eyes with > 25 mm Hg of IOP, which was statistically significant (p<0.001) concerning the other 2 non-myopic eyes that had an IOP of > 25 mm Hg. This could have been due to bio-mechanical stress induced by increased axial length and oxidative stress in myopic eyes [**9**]. We have considered central corneal thickness corrected IOP as all the myope eyes in our study had thinner corneas (spherical equivalent > -6D), which was not taken into consideration in previous studies. However, Park et al. [**6**] denied an association between high myopia and post-IVTA IOP spike. We observed 1 eye with a history of thyroidectomy (thyroid status not known) and was reported to have an IOP spike. However, due to the small sample size, such a correlation with the IOP spike could not be established [**23**]. 

We observed an IOP spike in 8 out of 30 phakic eyes, 4 out of 20 pseudophakic eyes, and 1 out of 10 aphakic eyes. We could not deduce any correlation with associated lens status as even in aphakic and pseudophakic eyes with compromised posterior capsule IOP spike was not significant when compared to phakic eyes. Park et al. [**6**] also observed normal IOP with a history of cataract surgery.

In our study, the cumulative incidence of high IOP was found in 21.7% of eyes, with more than 25 mm Hg in 6.7% of cases and more than 5 mm Hg rise from baseline IOP in 15% of eyes (p=0.62). However, 78.3% of eyes did not have any significant IOP fluctuations. Previous studies have reported 33% incidence and > 25 mm Hg IOP in 32% of eyes [**24**]. In our study, high baseline IOP itself lowered the threshold for high IOP to be reached easily, so we have considered more than 5 mm Hg rise from baseline as an IOP rise [**8**,**18**].

In our study, the pre-injection mean ± SD baseline IOP for uneventful eyes was 12.87 ± 2.65 and the pre-injection mean IOP for eyes with IOP event was 15.23 ± 2.89. The independent “t” test results showed that there was a significant difference in the mean of pre-injection baseline IOP concerning pre-injection mean IOP of eyes with IOP rise (t value=2.79, P=0.007*). Ahmad et al. [**24**] proposed the high baseline IOP as an independent risk factor for IOP spike following IVTA. Rhee DJ et al. [**25**] proposed that eyes with baseline IOP > 16 mm Hg should be monitored for an elevated IOP beyond 6 months.

Razeghinejad MR et al. [**2**] divided the normal population into 3 groups: 1) high responders - 4 to 6% who developed an IOP > 31 mm Hg or a rise of > 15 mm Hg above baseline; 2) moderate responders - 1/3rd population with IOPs 20-31 mm Hg or the rise of > 6-15 mm Hg; 3) non-responders - 2/3rd IOP < 6 mm Hg rise and < 20 mm Hg. 13 eyes in our study were moderate responders and the rest 47 were non-responders.

Raised IOP was present in 5% of eyes on day 1, 10% of eyes at 1 month, and 8.3% of eyes at 2 months. Our study reported a maximum IOP spike at 1 month (10%), followed by one at 2 months, which is comparable to the study of Kochabora et al. (17.7%) [**17**] and Cansu Yuksel-Elgin et al., who observed a maximum IOP at 1 month, while Karkhaya et al. reported a maximum IOP at 2.77 ± 3.72 months. In our study, the mean and standard deviation of time taken for IOP rise (in days) was 46.39 ± 37.68 or 1.53 ± 1.24 in months. Day 1 IOP spike could have been due to volume expansion as described by Lauer AK et al. [**9**], this being comparable with our study. We observed the mean IOP pre-injection to be 13.38 ± 2.85, with maximum IOP at 1-month follow-up 15.32 ± 4.10. In eyes with raised IOP, the IOP rise at each visit compared with the previous visit was significant (p=0.007). After adding AGMs (1 AGM in 12 eyes and 2 AGMs in 1 eye), target IOP was maintained throughout the study. IOP returned to baseline levels by the 5th and 6th month. TA maintains vitreous levels for up to 3 months [**5**-**7**] and causes chronic open-angle glaucoma by inducing physical and mechanical changes in trabecular meshwork by increased deposition of GAGs [**1**,**11**] and inhibiting proteases and trabecular meshwork endothelial cell phagocytosis [**1**,**2**,**11**]. So, IOP spikes by 1-2 months post IVTA. Similar trends of pre- and post-injection were reported by Balyen L et al. [**26**] and Ghoneim EM et al. [**27**].

*IVTA dose and IOP rise*

23.7% of 1 mg eyes and 18.2% of 2 mg eyes experienced an IOP rise. Apart from TA itself, certain associated factors triggered a higher incidence of IOP rise in the 1-mg group. However, in SCORE 15, Ahmad et al. [**24**] observed an IOP spike in only 5% of eyes in the 1 mg group and Lan-Hasin-Chuang et al. [**28**] reported 50% of eyes with raised IOP in the 2 mg group. In our study, the course of IOP in the 1 mg and 2 mg groups was not significant. The maximum IOP spike reached in the 1 mg arm was 24 mm Hg and in the 2 mg arm was 26 mm Hg. We observed the mean duration of the IOP spike to be higher in the 2-mg group - 46.89 ± 39.71 days, as compared to the 1-mg group - 45.25 ± 38.34 days, although this was not significant. The incidence of the IOP spike among the repeated dose of IVTA was 41.7%, which was more than the IOP spike among eyes with a single dose of IVTA - 16.7% (P=0.06). However, the maximum IOP spike with 1 dose of IVTA was 26 mm Hg, and with repeated IVTA it was 30 mm Hg. These eyes reported an IOP spike after the 2nd injection as repeated doses might have slowed the washout of TA from the vitreous. Lauer AK et al. [**9**] observed a 0.3% IOP spike in eyes with a single dose of IVTA and a 3% IOP spike with multiple injections.

Only 1 eye out of 13 with raised IOP showed IVTA crystals in the anterior chamber, which had received IVTA at 1 month and 2nd month for non-resolving CSME and associated an IOP spike of 30 mm Hg. 2 AGMs - Topical beta-blocker and alpha-adrenergic agonist were added, followed by the IOP reassessment on the 7th day. Repeated IVTA caused the spilling over of TA in the anterior chamber, but the rest of the eyes with repeated IVTA did not show this finding. Singh et al. [**14**] noted a significant rise in the IOP within 1 week of IVTA for refractory macular edema and deposition of white material in the angle on gonioscopy, which required surgical intervention to reduce the IOP.

We observed the mean BCVA pre-injection to be 0.68 ± 0.83 and improved to 0.46 ± 0.331 at 6 months, with no significant difference between groups as observed by Hauser et al. [**29**].

In the 1 mg group, pre-injection BCVA was 0.64 ± 0.35, which improved to 0.48 ± 0.36 at the end of 6 months. In the 2 mg group, pre-injection BCVA was 0.75 ± 1.32, which improved to 0.43 ± 0.29 at the end of 6 months.

The mean cup disc ratio pre-injection was 0.33 ± 0.10 and 0.33 ± 0.10 at 6 months. No significant changes related to disc were observed as the IOP spike was transient and well controlled with AGMs with frequent follow-up throughout the study. 

1 out of all 13 eyes with raised IOP needed 2 AGMs, the other 12 eyes responded well to 1 AGM. Although the mean duration of AGM use in the 2-mg group was 55.71 ± 32.07 days, which was more than that of the 1-mg group – 48 ± 26.83 days, this duration was not significant. However, previous studies by Karakhaya RH et al., and Ahmad et al. [**24**] have reported the need for surgery in refractory cases or in eyes not responding to maximum AGMs.

Various doses of IVTA have been described in literature like 1 mg, 2 mg, 4 mg, 20 mg, and so on, however, we compared the IOP effect on 1 mg and 2 mg and found that there were no differences in the IOP elevation. In our study, the risk factors were found to be high baseline IOP, repeated injection, and myopia.

Although the efficacy of 1 mg and 2 mg were not compared morphologically on ocular coherence tomography, considering that BCVA was comparable in both groups, we suggest that 2 mg IVTA can be used in recalcitrant ME, at the retinal physician’s discretion.

*Limitations*

The short follow-up period and the small sample size were limitations of the study. Also, all risk factors, type, and extent of lens changes, and central macular thickness were not observed and the number of letter improvements was not quantified.

## Conclusion

We propose that TA is an independent risk factor for post-intravitreal injection IOP spike. IVTA causes a maximum IOP spike at 1 to 2 months and has a protracted course that responds to AGMs. High baseline IOP, a repeated dose of IVTA, the presence of TA crystals in the anterior chamber, and high myopia were associated with significant IOP elevation. Moreover, it was found that 2 mg IVTA causes a longer duration of IOP spike and a longer duration of anti-glaucoma medications, hence, we recommend a lower dose of IVTA wherever it is feasible. However, a longer follow-up period would help in better risk factor analysis thereby permitting better individualization of the risk-benefit ratio for IVTA injection.

**Conflict of Interest Statement**

The authors state no conflict of interest.

**Informed Consent and Human and Animal Rights Statement**

Informed consent has been obtained from all individuals included in this study.

**Authorization for the use of human subjects**

Ethical approval: The research related to human use complies with all the relevant national regulations, and institutional policies, as per the tenets of the Helsinki Declaration, and has been approved by the review board of MM Joshi Eye Institue, Karnataka, India (Review letter No. IEC/MMJEI/DNB/2018/SEC 1, Dted- 5.11.2018).

**Acknowledgments**

We would like to thank Dr. Apoorva Ayachit and Dr. Priyanka Rane for their guidance.

**Sources of Funding**

The author(s) received no financial support for the research, authorship, and/or publication of this article.

**Disclosures**

None.
